# A randomized phase II trial of mitoxantrone, estramustine and vinorelbine or bcl-2 modulation with 13-cis retinoic acid, interferon and paclitaxel in patients with metastatic castrate-resistant prostate cancer: ECOG 3899

**DOI:** 10.1186/1479-5876-8-20

**Published:** 2010-02-24

**Authors:** Robert S DiPaola, Yu-Hui Chen, Mark Stein, David Vaughn, Linda Patrick-Miller, Michael Carducci, Bruce Roth, Eileen White, George Wilding

**Affiliations:** 1 Department of Medicine, The Cancer Institute of New Jersey, UMDNJ-RWJMS, New Brunswick NJ, USA; 2 Department of Biostatistics, Dana Farber Cancer Center, Harvard, Boston MA, USA; 3 Department of Medicine, University of Pennsylvania, Philadelphia PA, USA; 4 Department of Medicine, Johns Hopkins University, Baltimore, MD, USA; 5 Department of Medicine, Vanderbilt University, Nashville, Tennessee, USA; 6 Department of Molecular Biology and Biochemistry, Rutgers University, Piscataway, NJ, USA; 7 Department of Medicine, University of Wisconsin, Madison, WI, USA

## Abstract

**Background:**

To test the hypothesis that modulation of Bcl-2 with 13-cis retinoic acid (CRA)/interferon-alpha2b (IFN) with paclitaxel (TAX), or mitoxantrone, estramustine and vinorelbine (MEV) will have clinical activity in men with metastatic castrate-resistant prostate cancer (CRPC).

**Methods:**

70 patients were treated with either MEV (Arm A) in a 3-week cycle or CRA/IFN/TAX with an 8-week cycle (Arm B). Patients were assessed for response, toxicity, quality of life (QOL), and the effect of treatment on Bcl-2 levels in peripheral blood mononuclear cells (PBMC).

**Results:**

The PSA response rates were 50% and 23%, measurable disease response rates (CR+PR) 14% and 15%, and median overall survival 19.4 months and 13.9 months on Arm A and Arm B respectively. Transient grade 4 neutropenia occurred in 18 and 2 patients, and grade 3 to 4 thrombosis in 7 patients and 1 patient in Arm A and Arm B respectively. Patients on Arm B reported a clinically significant decline in QOL between baseline and week 9/10 (.71 s.d.), and a significantly lower level of QOL than Arm A (p = 0.01). As hypothesized, Bcl-2 levels decreased with CRA/IFN therapy only in Arm B (p = 0.03).

**Conclusions:**

Treatment with MEV was well tolerated and demonstrated clinical activity in patients with CRPC. Given the adverse effect of CRA/IFN/TAX on QOL, the study of other novel agents that target Bcl-2 family proteins is warranted. The feasibility of measuring Bcl-2 protein in a cooperative group setting is hypothesis generating and supports further study as a marker for Bcl-2 targeted therapy.

**Trial Registration:**

**Clinical Trials Registration number**: CDR0000067865

## Background

It was estimated that approximately 200,000 new patients were diagnosed with prostate cancer, and 40,000 died from their disease in 2008 [1]. Standard hormonal therapy or chemotherapy is limited in effectiveness against metastatic prostate cancer because of the development of tumor resistance. Options for improved survival in patients with castrate-resistant prostate cancer (CRPC) are limited with only data clearly established for the use of docetaxel chemotherapy [2].

An important mechanism of tumor resistance, which can be exploited therapeutically, is the over-expression of Bcl-2. The over-expression of Bcl-2 is implicated as a cause of hormonal and chemotherapy resistance and has been shown to increase with castration in prostate cancer [3,4]. Prior studies conducted by our group and other investigators demonstrated that retinoids can decrease expression of Bcl-2, that the combination of 13-cis retinoic acid (CRA) and interferon (IFN) enhanced the effect of paclitaxel chemotherapy, and that the combination can be safely administered in phase I studies [5-7]. More recently, our group and other investigators have studied novel BH3 domain mimetics such as AT101 and ABT263, which modulate multiple Bcl-2 family proteins, and are being tested in early clinical studies [8-10].

The development of novel chemotherapeutic combinations to overcome tumor resistance may also be important [11,12]. In this regard, previous data demonstrated activity with estramustine-based combinations and the activity of combined navelbine and mitoxantrone, supporting the testing of combination therapy with estramustine, mitoxantrone, and vinorelbine in patients with CRPC. One study demonstrated a potential clinical benefit to adding estramustine with vinorelbine as second line therapy in CRPC [13]. Another study conducted by the Hellenic Cooperative Oncology Group, evaluated the safety and activity of the combination in CRPC and found evidence of activity including complete responses in measurable disease [14].

Clearly, further efforts are needed to understand and target mechanisms of tumor resistance. In the current study, two regimens are tested in a randomized phase II selection design study in an attempt to develop various approaches to abrogate resistance in CRPC. Therefore, the results of this study using CRA/IFN with weekly paclitaxel (Arm B of this randomized phase II study) may be helpful to this area of drug development to determine the activity of the combination, and to understand peripheral blood mononuclear cell Bcl-2 protein expression as a potential biomarker for future studies. Given the possibility of estrogen effect on Bcl-2 expression, arm A of our study was conducted to both confirm the activity of the combination of estramustine, mitoxantrone and vinorelbine, as well as to assess the effect on Bcl-2 protein in peripheral blood mononuclear cells, as planned for Arm B of the study. Additionally, patients with CRPC are at risk of replacing disease related symptoms with treatment associated symptoms. Thus, evaluation of patients' symptoms and QOL was an important endpoint in the study [15].

## Methods

### Patients

To be eligible for this study, patients were required to have an ECOG performance status of 0, 1, or 2, have histologically proven adenocarcinoma of the prostate gland, and evidence of progressive metastatic disease within 4 weeks of study entry. Patients with an elevated serum alkaline phosphatase or PSA level as the only evidence of disease were ineligible. Patients with only bone metastases were required to have a PSA level of ≥ 20 ng/ml. Patients with soft tissue metastases and/or visceral disease were required to have either measurable disease or a PSA level of ≥ 20 ng/ml. Patients were required to have prior treatment with bilateral orchiectomy or other primary hormonal therapy with evidence of treatment failure (patients who had not undergone bilateral orchiectomy were required to continue LHRH agonist therapy while receiving protocol therapy). Flutamide or Nilutamide must have been discontinued at least 4 weeks prior to randomization, and bicalutamide at least 6 weeks prior to randomization, with evidence of progressive disease. Patients could not have had prior chemotherapy or prior Strontium 89, Samarium 153, or other radioisotope therapies. Patients must have had adequate bone marrow function, defined as WBC ≥ 4000/mm^3^, granulocytes ≥ 2000/mm^3^, platelet count ≥ 100,000/mm^3^, bilirubin ≤ 1.5 mg/dl, SGOT (AST) and SGPT (ALT) ≤ 2 times the institutional upper limit of normal, creatinine ≤ 2.0 mg/dl or a calculated creatinine clearance ≥ 50 ml/min, LVEF ≥ 50% as proven by MUGA within 4 weeks of study entry, no active angina pectoris, or known heart disease of New York Heart Association Class III-IV. Patients must have had no history of myocardial infarction within 6 months of study entry and no history of deep venous thrombosis. The requirement of MUGA evaluation for LVEF was added in Addendum 2 (December 2001).

### Study Design

This study was conducted by the Eastern Cooperative Oncology Group (ECOG), required institutional review board approval at each ECOG institution, and required an informed consent for each patient. Patients were randomly assigned to either Arm A or B. Patients randomized to Arm A received vinorelbine 25 mg/m^2 ^intravenously on days 2 and 9, followed by mitoxantrone 10 mg/m^2 ^intravenously on day 2 and estramustine 280 mg orally twice a day on days 1 through 5 of a 3-week cycle. Patients were continued on treatment until evidence of disease progression, unacceptable toxicity or to a cumulative mitoxantrone dose of 140 mg/m^2^. Patients with prior pelvic irradiation were treated with a 25% dose reduction of mitoxantrone (reduced dose = 7.5 mg/m^2^). Patients randomized to Arm B received 13-cis retinoic acid (CRA) 1 mg/kg orally, and interferon-alpha2b (IFN) 6 MU/m^2 ^subcutaneously, each administered on days 1 and 2 of each week, followed by paclitaxel 75 mg/m^2 ^intravenously on day 2 of each week with an 8-week cycle (Arm B). Each 8-week course consisted of 6 weeks of treatment followed by a 2-week rest. Treatment was continued until evidence of disease progression or unacceptable toxicity. As prophylaxis for hypersensitivity reactions, patients were premedicated with dexamethasone 20 mg, cimetidine (or ranitidine) 300 mg (50 mg), and benadryl 50 mg intravenously 30 minutes prior to each dose of paclitaxel. All dosing was based on patients' actual weight at the beginning of each cycle. Conditions for dose modification in the case of toxicity were specified in the protocol.

### Efficacy and Safety

Response and progression were assessed according to RECIST and were determined by investigator assessment of radiographs and PSA. All toxicities were graded according to the Common Toxicity Criteria (CTC) version 2.0. Three self-reported measures of QOL were completed by patients in both arms: the Functional Assessment of Cancer Therapy, Prostate Cancer Version 4 (FACT-P), the Brief Pain Inventory- Short Form (BPI), and the Schwartz Cancer Fatigue Scale (SCFS). FACT-P consists of 27 core items, which assess patient function (FACT-G) and a 12-item prostate cancer subscale (PCS) to assess symptoms and problems specific to prostate cancer. In addition, the 26-item Trial Outcome Index (TOI) was computed by combining the physical well-being, functional well-being, and PCS subscales. Pain intensity and interference with functioning were assessed with the BPI. All patients completed the QOL assessment at baseline. QOL was also assessed for Arm A patients on day 2 of cycles 2, 4, and 6 as well as completion of treatment; for Arm B patients, on day 22 of cycle 1 and day 1 of cycles 2 and 3 and completion of treatment. In the case of missing responses in the FACT measures, if at least half of the subscale items were answered, the subscale score was the average of the completed items multiplied by the number of items in the subscale. Item responses are averaged across the BPI to indicate pain intensity and interference. Higher scores for BPI indicate worse pain or more interference; higher scores for the FACT and SCFS indicate better quality of life.

### Bcl-2 assessment

Peripheral blood was collected on day 1 and day 3 of the first cycle of treatment to analyze mononuclear cell Bcl-2 levels, as previously described [5]. An actin control was used to adjust for the differences in the amount of protein loaded. A comparison between the Bcl-2 levels on day 1 and day 3 of the first cycle was done to determine the effect of protocol treatment on Bcl-2 levels. A patient with a 50% or greater decrease in western band intensity on densitometer measurement was considered to have a Bcl-2 response.

### Statistical Design and Data Analysis

Patients were equally randomized to either of the two treatment regimens and stratified by extent of disease (measurable disease vs. non-measurable disease and elevated PSA). The primary endpoint was the proportion of patients responding by 6 months. PSA response was defined as PSA decline from baseline value by ≥ 50%, or normalization of PSA (<0.2 ng/mL), confirmed by a second measurement at least 1 week later. An underlying true response rate of 40% was considered evidence that the treatment merited further study. On the other hand, an underlying true response rate of 10% would be of no clinical interest.

A two-stage accrual plan was employed for this study. First, 7 eligible patients per arm were to be registered to the study and assessed for PSA response at 6 months. Accrual would continue while these patients were followed for the primary endpoint. Upon assessment, if 1 or more of the 7 eligible patients responded, accrual would continue to 30 eligible patients (35 total) per arm. If none of the first 7 eligible patients demonstrated response, the study would halt and the other patients accrued by this time would be assessed. A stochastic curtailment algorithm was used to guide decision-making. For each potential number of patients registered at the time response assessment among the first 7 eligible patients was complete, the number of stage 1 responses required to reopen accrual (out of all patients enrolled at that time) was identified in the protocol, based on an ad hoc rule, along with the corresponding probability of stopping early under the null hypothesis (response rate of 10%), and the probabilities of rejecting the treatment under the null and alternative hypotheses. If 7 or more of the 30 eligible patients responded by 6 months, we would conclude that the treatment warranted further study. The probability of concluding that the treatment was effective was at least 90 to 98% if the true response rate was 40%. The probability of stopping early if the treatment was ineffective (true response rate of 10% or less) was at least 48% and might be as high as 94%. If 7 responses were observed among the 30 eligible patients, the two-stage 90% confidence interval would be approximately 12% to 41%. If the total number of responses among the 30 eligible patients was 7 or more on a given arm, additional patients with measurable disease would be enrolled, such that the total number of patients with documented measurable disease was 27 per arm, of whom 25 were eligible. Following this second phase of accrual, the response rates among patients with and without measurable disease and their respective 90% confidence intervals would be computed. The 90% confidence interval around the true response rate among eligible patients with measurable disease would be no wider than 36%.

Survival time was defined as the time from study entry until death or date last known alive. Progression-free survival was defined as the time from registration to first progression of any applicable type (measurable disease, PSA, or bone) or death, whichever came first (for patients who progressed or died), or time from registration until date last known progression-free (for patients who are alive without progression). For patients with measurable disease who did not progress, time to progression was censored at the last measurable disease assessment or bone scan, whichever came first. For patients without measurable disease who did not progress, time to progression was censored at the last bone scan or last PSA measurement, whichever came first.

Descriptive statistics were used to characterize patients at study entry. Patient demographics, adverse events, and response rates were compared using Fisher's exact test. The method of Kaplan and Meier was used to characterize overall survival and progression-free survival. The stratified log rank test was used to test for differences in overall survival and progression-free survival by treatment. The Wilcoxon signed-rank test was used to test the differences in Bcl-2 levels between Day 1 Cycle 1 and Day 3 Cycle 1. All p-values are two sided.

In order to evaluate the effects of treatment on QOL, changes in the FACT-P, TOI, BPI, and SCFS from baseline to week 9/10 were calculated for each patient, and evaluated using the Wilcoxon signed-rank test. Three patients with measurable disease were stratified incorrectly to the non-measurable disease and elevated PSA stratum (2 Arm A patients and 1 Arm B patient), while 1 Arm A patient without measurable disease was stratified incorrectly to the measurable disease stratum. All patients were analyzed according to their actual extent of disease regardless of how they were stratified.

## Results

### Patient Characteristics

The study was activated on January 31, 2001 and completed on October 7, 2003 after reaching its accrual goal of 70 patients. Eighteen main ECOG institutions contributed patients to the study. Seven patients were ineligible, and sixty-three patients were included in the main analysis. Patient demographic factors and disease characteristics of the eligible patients at study entry are provided in Table 1. Overall, the median age was 68 years with a range of 45 to 89 years. Most patients (95%) had ECOG Performance Status of 0 or 1. About 90% of patients were Caucasian. All patients had metastatic disease, most commonly in the bone (90%). Ninety-five percent had been treated with hormonal therapy, and 27% had prior orchiectomy. As shown in Table 1, baseline demographic and tumor characteristics were generally well balanced between the two arms, and no significant difference between the two arms was found.

**Table 1:** Patient Characteristics

	Arm A (n = 32)		Arm B (n = 31)		Total (n = 63)	
	
	n	Percent	n	Percent	n	Percent
Age						
Median		70		65		68
Range		50-89		45-80		45-89
Race						
White	30	94%	28	90%	58	92%
Black	1	3%	3	10%	4	6%
Hispanic	1	3%	0	0%	1	2%
ECOG PS						
0	15	47%	17	55%	32	51%
1	16	50%	12	39%	28	44%
2	1	3%	2	6%	3	5%
Extent of Disease						
Measurable, Non-Osseous Disease Present	14	44%	13	42%	27	43%
Evaluable, Osseous Disease Present	28	88%	30	97%	58	92%
Evaluable, Non-Osseous Disease Present	13	41%	12	39%	25	40%
Elevated PSA	32	100%	30	97%	62	98%
Metastatic Disease						
Lung	4	13%	4	13%	8	13%
Liver	3	9%	3	10%	6	10%
Bone	28	88%	29	94%	57	90%
Bone Marrow	0	0%	1	3%	1	2%
Pleura	1	3%	0	0%	1	2%
Other	11	34%	9	29%	20	32%
Prior Treatment						
Orchiectomy	10	31%	7	23%	17	27%
Prostatectomy	17	53%	15	48%	32	51%
Other Surgery	19	59%	20	65%	39	62%
Radiation Therapy	18	56%	20	65%	38	60%
Hormonal Therapy	31	97%	29	94%	60	95%
Biologic Response Modifier	4	13%	2	6%	6	10%

### Adverse Events

Patients received a median of 5 and 2 cycles (15 and 16 weeks total) of protocol therapy on Arm A, which used 3-week cycles, and Arm B, which used 8-week cycles, respectively. Forty-one percent were off treatment due to progressive disease. Information about symptoms and toxicities was collected at baseline as well as during and following treatment. All patients who received protocol therapy, regardless of eligibility, were evaluated for toxicities. Table 2 shows toxicities experienced by patients during therapy. Hemoglobin was the most frequently occurring toxicity. Nineteen and five patients experienced life-threatening toxicities on Arm A and Arm B, respectively, and the most frequently observed Grade 4 toxicity was neutropenia. The proportion of patients with Grade 3 or higher toxicity was significantly higher on Arm A for leukocytes (p < 0.001), neutrophils (p < 0.001), and worst degree toxicity (p = 0.004).

**Table 2:** Treatment-Related Toxicities (Patient Number)

	Arm A (n = 35)			Arm B (n = 35)				Arm A (n = 35)			Arm B (n = 35)		
					
	Grade			Grade				Grade			Grade		
					
Toxicity Type	1, 2	3	4	1, 2	3	4	Toxicity Type	1, 2	3	4	1, 2	3	4
Allergic reaction	-	-	-	-	2	-	Stomatitis	5	-	-	6	1	-
Allergic rhinitis	1	-	-	1	-	-	Taste disturbance	6	-	-	4	-	-
Hemoglobin	31	3	1	28	5	-	Vomiting	10	-	-	9	1	-
Leukocytes	9	18	8	22	4	2	Diarrhea	5	2	-	11	-	-
Neutrophils	4	11	18	16	7	2	GI-other	2	-	-	-	-	-
Platelets	20	5	-	13	1	-	Hematuria	-	1	-	1	-	-
Transfusion:	-	1	-	-	4	-	Rectal bleeding	-	-	-	-	1	-
Sinus tachycardia	-	1	-	-	1	-	Alkaline phos	13	2	-	15	3	-
Supraventricular	-	-	-	-	1	-	Bilirubin	1	1	1	5	-	-
Cardiac-ischemia	-	-	-	1	-	-	Hypoalbuminemia	-	-	-	1	-	-
Cardiac-left vent	-	1	-	-	-	-	SGOT	7	-	-	10	1	-
Edema	9	-	-	4	-	-	SGPT	6	-	-	7	1	-
Hypertension	2	-	-	1	-	-	Febrile neutropenia	-	4	-	-	1	-
Hypotension	1	-	-	3	-	-	Infection w/neutro	-	1	1	-	-	-
Phlebitis	1	-	-	-	-	-	Infectionw/oneutro	2		-	1	1	-
Thrombosis/emb	-	6	1	-	1	-	Hypercalcemia	1	-	-	1	-	-
Fatigue	21	7	1	23	7	1	Hyperglycemia	2	1	-	3	1	-
Fever	-	-	-	7	1	-	Hyperkalemia	1	-	-	1	-	-
Rigors/chills	1	-	-	10	-	-	Hypertrig	6	-	-	22	1	-
Sweating	-	-	-	2	-	-	Hyponatremia	1	-	-	1	-	-
Weight gain	1	-	-	-	-	-	Metabolic-other	1	-	-	-	-	-
Weight loss	14	-	-	7	2	-	Muscle weakness	1	-	-	-	1	-
PTT	-	1	-	-	-	-	Ataxia	-	-	-	1	-	-
PT	1	1	-	-	-	-	Dizziness	3	-	-	3	-	-
Alopecia	8	-	-	11	-	-	Hallucinations	-	-	-	-	1	-
Bruising	2	-	-	-	-	-	Insomnia	2	-	-	7	-	-
Dry skin	1	-	-	3	-	-	Depression	4	1	-	2	-	-
Flushing	-	-	-	3	-	-	Euphoria	1	-	-	-	-	-
Injection site	2	-	-	-	-	-	Neuropathy-motor	2	-	-	1	-	-
Pruritus	2	-	-	-	-	-	Neuro-sensory	6	-	-	9	-	-
Rash	4	-	-	2	-	-	Vertigo	1	-	-	-	-	-
Skin-other	2	-	-	-	-	-	Neurologic-other	1	-	-	-	-	-
Gynecomastia	3	-	-	-	-	-	Tearing	1	-	-	-	-	-
Hot flashes	3	-	-	1	-	-	Blurred vision	1	-	-	3	-	-
Endocrine-other	-	-	-	1	-	-	Abdominal pain	2	-	-	-	-	-
Anorexia	16	1	-	14	2	-	Arthralgia	2	-	-	3	-	-
Constipation	12	1	-	8	-	-	Bone pain	1	-	-	5	-	-
Dehydration	-	1	-	2	1	-	Chest pain	1	-	-	-	-	-
Dyspepsia	3	-	-	-	-	-	Headache	2	-	-	8	-	-
Dysphagia	1	-	-	1	-	-	Myalgia	4	-	-	11	-	-
Flatulence	1	-	-	1	-	-	Pain-other	2	-	-	-	-	-
Mouth dryness	-	-	-	3	-	-	Cough	5	-	-	-	-	-
Nausea	21	-	-	18	1	-	Dyspnea	7	-	1	11	1	-
Sense of smell	1	-	-	-	-	-	Hypoxia	-	-	-	-	1	-
Creatinine/GU	8	-	1	3	-	2	Pulmonary-other	-	-	-	3	1	-

### Clinical and Biochemical Effect

The PSA response rates were 50% (90% CI: [34%, 66%]) on Arm A and 23% (90% CI: [11%, 38%]) on Arm B among eligible patients. There were 16/32 and 7/31 responses on Arm A and Arm B, respectively. Although the primary objective was not to compare the two treatment arms, an additional intent-to-treat analysis of response demonstrated a 51% (18/35) response rate in arm A and 20% (7/35) response rate in arm B. Sixteen patients were unable to be evaluated for PSA response. Of the 63 eligible patients, 14 and 13 had measurable disease at study entry on Arm A and Arm B, respectively. Six patients were unable to be evaluated for objective response. In patients with measurable disease, no CR and 2 PRs were observed on both arms. The overall response rates (CR+PR) among patients with measurable disease were 14% on Arm A and 15% on Arm B.

**Figure 1:**
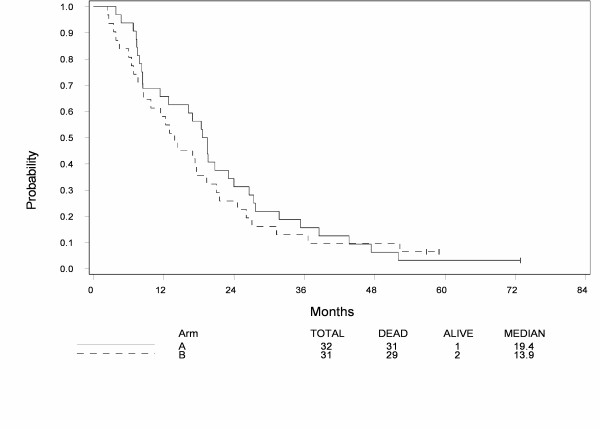
**Overall Survival in patients treated with mitoxantrone, estramustine and vinorelbine (Arm A) or 13-cis retinoic acid, interferon-alpha2b with paclitaxel (Arm B)**.

**Figure 2:**
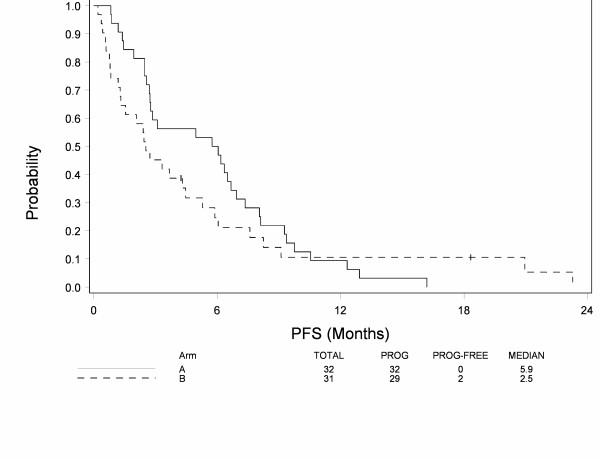
**Progression Free Survival in patients treated with mitoxantrone, estramustine and vinorelbine (Arm A) or 13-cis retinoic acid, interferon-alpha2b with paclitaxel (Arm B)**.

At the time of analysis, 60 patients have died and 3 patients are still alive among eligible patients. Median follow-up among patients still alive is 59 months. Figure 1 shows overall survival by treatment. Median survival is 19.4 months for Arm A and 13.9 months for Arm B. There was no statistically significant difference in overall survival by treatment while controlling for extent of disease (p = 0.42), but the study was not powered to detect such difference. Figure 2 shows progression-free survival by treatment. Median progression-free survival is 5.9 months for Arm A and 2.5 months for Arm B. There was no statistically significant difference in progression-free survival across treatments after adjusting for extent of disease (p = 0.34), but the study was not powered to detect such difference.

### Bcl-2 Levels

Among the 63 eligible patients, 18 and 16 patients had Bcl-2 levels available on Arm A and Arm B, respectively. For Arm A, the average Bcl-2/actin ratio was 0.88 and 1.32 on day 1 and day 3 of cycle 1, respectively, and the difference was not significant (p = 0.47). For Arm B, the average Bcl-2/actin ratio was 1.55 and 1.00 on day 1 and day 3 of cycle 1, respectively, and the difference was statistically significant (p = 0.03). Although the number assessed was small, no significant association was found between Bcl-2 response and PSA response on either arm.

### Quality of Life

**Figure 3:**
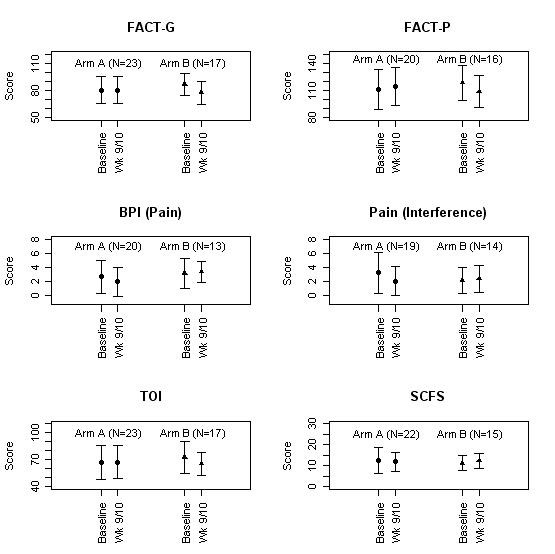
**Changes in quality of life from baseline to week 9/10 (day 2 of cycle 4 for Arm A and day 1 of cycle 2 for Arm B)**.

The primary QOL outcomes were assessed by the FACT-P and the TOI. Changes from baseline to week 9/10 (day 2 of cycle 4 for Arm A and day 1 of cycle 2 for Arm B) are shown in Figure 3. In Arm A the change in QOL measures (FACT-P and TOI) did not differ significantly from baseline to week 9/10; the average change in FACT-P (mean = 3.5; sd = 18.6) did not represent a clinically meaningful difference between baseline and week 9/10. For patients in Arm B, FACT-P (p = 0.01) and TOI (p = 0.02) were significantly lower at week 9/10 than baseline (mean = -9.8; 0.71 s.d.). Changes in symptom measures (BPI and SCFS) were non-significant in both arm A and Arm B; however, the study was not powered to detect statistical significance in these measures.

## Discussion

We found that treatment with vinorelbine, mitoxantrone and estramustine yielded a response rate suggesting clinical activity in men with CRPC, and was well tolerated. The combination of CRA/IFN and paclitaxel, in contrast, resulted in clinically meaningful decline in QOL, warranting the study of other novel agents that target Bcl-2 family proteins. Effects of therapy on PBMC Bcl-2 protein supports the feasibility of measuring Bcl-2 family proteins in a multi-institution cooperative group setting.

The treatment of CRA/IFN and paclitaxel was designed to modulate Bcl-2 mediated drug resistance based on studies demonstrating that Bcl-2 over-expression is implicated as a cause of hormonal and chemotherapy resistance in prostate cancer, and prior studies demonstrating an effect of CRA/IFN on Bcl-2 regulation [3-6]. We found a modest PSA response rate of 23% and a progression free survival of only 2.5 months. Potential reasons for such modest activity may include the lack of effect by CRA/IFN, the choice of taxane, or limited therapy due to excessive toxicity. As shown in Figure 3, patients had significantly lower QOL scores, consistent with a clinically meaningful decline in QOL at week 9/10 compared with baseline [16]. However, Bcl-2/actin ratios decreased in PBMCs with therapy, supporting further study of this as a marker of drug effect with Bcl-2 modulating therapies. In fact, our center and other investigators are now developing novel agents such as the BH3 domain mimetics, which modulate multiple Bcl-2 family proteins [8-10].

The treatment arm with vinorelbine, mitoxantrone and estramustine was designed based on data demonstrating activity of this combination in limited studies. We found a PSA response rate of 50%, progression free survival of 5.9 months, and overall survival of 19.4 months, consistent with clinical activity of this regimen. This is in agreement with a prior study of this regimen, which demonstrated a 56% PSA response, median duration of response of 6.9 months, and survival of 14.5 months [14]. Although this regimen was administered as a first line therapy in patients without prior chemotherapy, further studies of this regimen in patients that have progression after docetaxel therapy may be useful because docetaxel is approved first line therapy in hormone refractory prostate cancer.

## Conclusions

Treatment with MEV was well tolerated and could be studied further as first line therapy, or as a second line therapy. Given the adverse effect of CRA/IFN/TAX on QOL, the study of other novel agents that target Bcl-2 family proteins is warranted. The feasibility of measuring Bcl-2 protein in a cooperative group setting is hypothesis generating and supports further study as a marker for Bcl-2 targeted therapy.

## Competing interests

The authors declare that they have no competing interests.

## Authors' contributions

RSD, YC, MS, MC, BR, EW, and GW contributed to enrollment, conception, design, interpretation of data and reading and approval of the final manuscript. LPM completed analysis and interpretation of quality of life data and approved of the final manuscript. DV contributed to enrollment, interpretation of data and reading and approval of the final manuscript. YC also performed all statistical analysis.
